# Shifting paradigms: how the glenoid track changed the concept of shoulder instability

**DOI:** 10.1007/s00264-026-06930-z

**Published:** 2026-06-30

**Authors:** Eiji Itoi, Nobuyuki Yamamoto

**Affiliations:** 1 Tohoku Rosai Hospital, Sendai, Japan; 2 Tohoku University, Sendai, Japan

**Keywords:** Shoulder instability, Bony lesions, Glenoid bone loss, Hill-Sachs lesion, Glenoid track

## Abstract

**Background:**

Historically, glenoid bone loss and humeral head defects (Hill-Sachs lesions) were evaluated independently. However, the clinical significance of a Hill-Sachs lesion depends on the morphology of the glenoid: an identical lesion may remain stable with an intact glenoid but become unstable in the presence of glenoid bone loss. Therefore, both lesions must be assessed simultaneously.

**Limitation of traditional assessment:**

Traditional dynamic intraoperative examination has an inherent limitation. Engagement can be accurately assessed only after Bankart repair (post-repair examination), whereas remplissage, when required, must be performed before the repair (pre-repair examination).

**Introduction of a new concept:**

To overcome this dilemma, the ‘glenoid track’ concept was introduced, enabling combined assessment of Hill-Sachs lesions and glenoid bone loss. Since its introduction, the concept has evolved through refinements such as consideration of subcritical glenoid bone loss, subdivision into central and peripheral tracks, and patient-specific adjustment of track width based on the range of motion. Efforts are also underway to establish accurate MRI-based assessment, facilitating widespread radiation-free application.

**Extension of the concept:**

Furthermore, the concept has been extended to posterior shoulder instability through the development of the ‘reverse glenoid track’ concept, broadening its applicability across the spectrum of shoulder instability.

## The pre-track era

Bony lesions of the glenoid and the humeral head are very common in shoulders with anterior instability. In recurrent shoulder dislocations, the glenoid bone loss is observed in 74–86%, Hill-Sachs lesion in 80–94%, and the combination of these lesions known as a bipolar lesion is observed in 62–81% [1, 2].

The glenoid bone loss is classified into two types: fragment type and erosion type [3]. The erosion type refers to a bone loss without a bony fragment. It was named “erosion” because this type of bony defect was thought to be the result of recurrent dislocations. However, this type of bone loss is sometimes seen after a first-time dislocation. A cadaveric study showed that the erosion type bone loss was created after a single dislocation and the histology showed a compression fracture of the glenoid rim [4]. Because of these findings, it is considered more appropriate to call this type of bone loss the “compression type” rather than the “erosion type”.

What is wrong with the glenoid bone loss? In our previous study, we created a glenoid bone loss stepwise at the anteroinferior rim of the glenoid and checked the stability [5]. When the glenoid bone loss exceeded 21% of the glenoid length, the shoulder stability could not be achieved even after the Bankart repair [5]. Later, we found that the glenoid bone loss was located almost anterior to the glenoid [6]. Subsequent biomechanical studies revealed that the anterior glenoid bone loss also caused shoulder instability when it exceeded 25% of the glenoid width [7, 8]. The clinical data also showed that the critical size of the glenoid defect was 20–25% [9, 10]. However, as the transition between the critical and safe zones was not distinct, recent studies have defined an intermediate gray zone known as subcritical bone loss, with reported ranges spanning 13.5–20% [11] and 17–25% [12]. These discrepancies are likely attributable to differences in the risk profiles of the respective study cohorts.

The glenoid bone loss is associated with mid-range instability, and its risk of inducing instability is independent of a Hill-Sachs lesion [13]. Therefore, the aforementioned critical and subcritical bone loss thresholds can be utilized as stand-alone criteria in clinical practice.

**Fig. 1:**
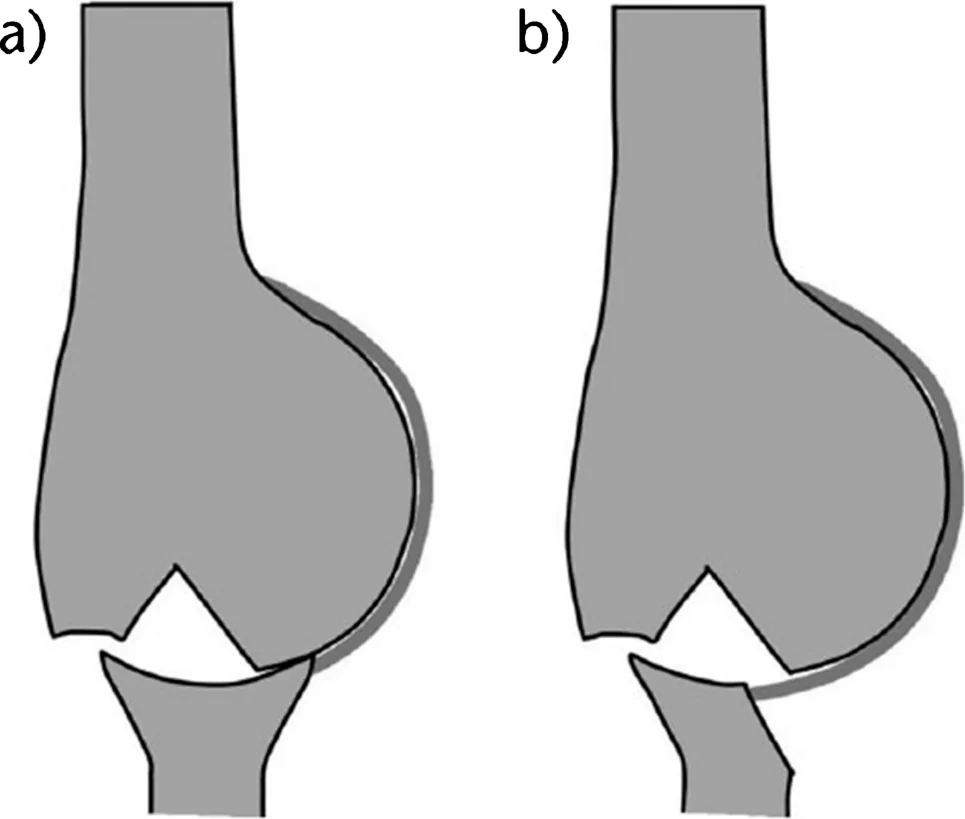
Hill-Sachs lesion and the glenoid. **a** The shoulder remains stable at the end-range of motion if a Hill-Sachs lesion is entirely contained within the intact glenoid surface. **b** However, the same lesion leads to recurrent instability when coupled with a glenoid bone loss; the compromised glenoid width fails to span the defect, allowing the lesion to engage and cause a dislocation. (Reprinted from Fig. 14, Itoi [13])

The risk assessment of Hill-Sachs lesion, however, is much more complex. While a shoulder is stable if a Hill-Sachs lesion is fully covered by the glenoid at the end-range of motion, the same lesion can cause instability when coupled with a glenoid bone loss (Fig. 1). Consequently, the risk of a Hill-Sachs lesion is dependent on the morphology of the glenoid [13]. Therefore, both the glenoid and the Hill-Sachs lesion should be assessed simultaneously. Nevertheless, previous literature on the surgical indications for a Hill-Sachs lesion has focused exclusively on the lesion itself—quantifying merely its area [14–16], depth [17], or volume [17]—while completely overlooking the concomitant role of the glenoid.

## Scientific genesis

To address the limitations of the pre-track era, a dynamic examination evaluating the engagement between the anterior glenoid rim and the Hill-Sachs lesion might appear to be a viable solution. However, such examinations are typically performed prior to Bankart repair. With the Bankart lesion left unfixed, the resulting anterior instability allows the humeral head to displace anteriorly, causing the Hill-Sachs lesion to engage prematurely and potentially leading to recurrent dislocation. Consequently, pre-repair assessment fails to accurately reflect the true engagement risk. To address this, the dynamic examination should ideally be performed after Bankart repair. Yet, a post-repair examination carries two distinct risks: first, overstretching may compromise the newly repaired tissue, and second, it introduces technical challenges for any subsequent remplissage procedure.

If a true engagement risk is identified, a remplissage—involving tenodesis of the infraspinatus tendon into the Hill-Sachs lesion—is indicated. However, performing a remplissage after Bankart repair is technically demanding; once the humeral head is properly centered within the glenoid socket, its reduced anterior translation hinders both bony bed preparation and suture anchor insertion within the Hill-Sachs defect. Therefore, from a technical standpoint, preparation must precede Bankart repair, whereas a precise dynamic assessment requires Bankart repair to precede the examination. These two clinical requirements are fundamentally contradictory and mutually exclusive. This clinical dilemma underscores why a dynamic examination cannot serve as a definitive solution. Indeed, when evaluated post-repair, the true prevalence of engaging Hill-Sachs lesions was only 7.1% [18], markedly lower than the 34–46% reported by studies utilizing pre-repair examinations [19–21]. To resolve this paradox, we introduced the novel concept of the 'glenoid track,' which enables the precise, non-invasive prediction of true engaging Hill-Sachs lesions preoperatively.

As illustrated in Fig. 1, the shoulder remains stable if the Hill-Sachs lesion is fully covered by the glenoid, whereas stability is compromised if the lesion is not completely covered by the glenoid at the end-range of motion. Consequently, clarifying the precise relationship between the Hill-Sachs lesion and the glenoid was imperative. The initial step required characterizing the translation of the glenoid relative to the humeral head when the arm was positioned in anterior apprehension [22], along the posterior end-range of motion. In this position, if the Hill-Sachs lesion is entirely covered by the glenoid (Fig. 1a), the shoulder remains stable; if not (Fig. 1b), recurrent dislocation can occur.

**Fig. 2:**
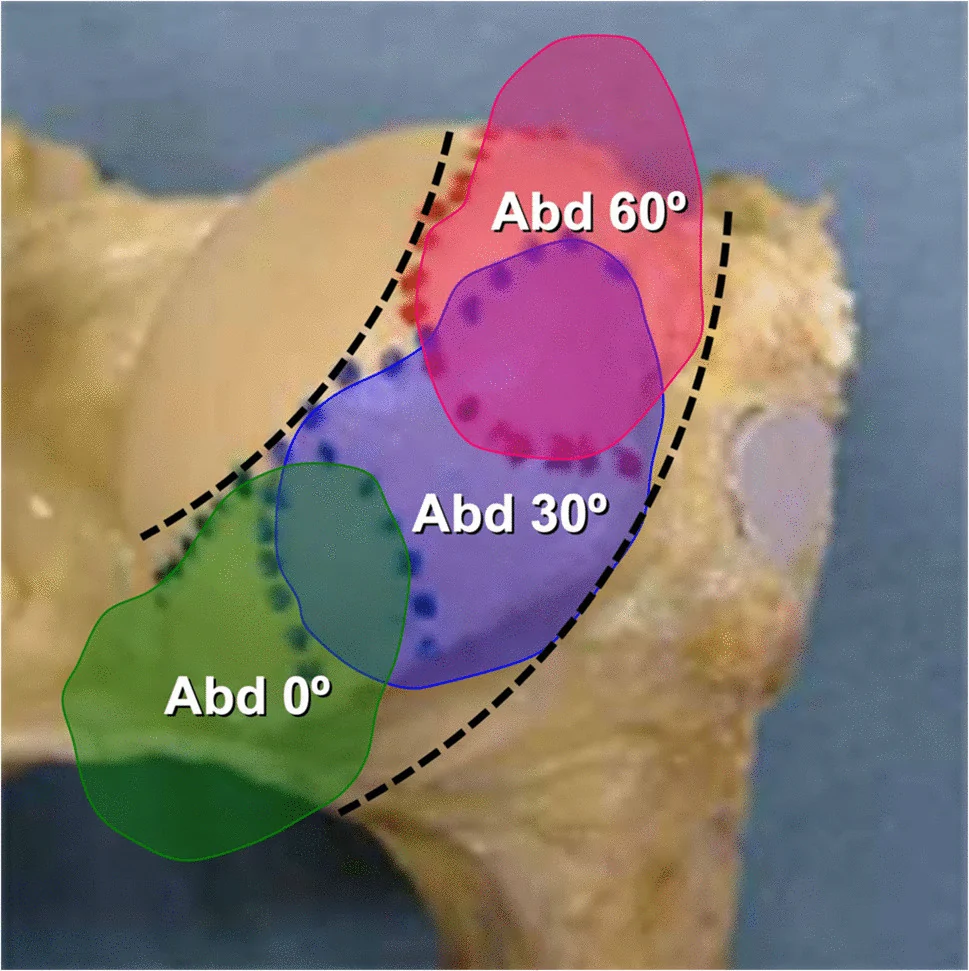
Glenoid track in a cadaveric shoulder. The glenoid moved along the posterior margin of the articular surface of the humeral head as the arm was moved from abduction 0° up to 60°. (Reproduced with modification from Fig. 4 A, Yamamoto et al. [23])

This kinematic relationship between the glenoid and the humeral head was elucidated in our initial cadaveric study [23]. After removing all the soft tissues except for the joint capsule, the arm was positioned at 0°, 30°, and 60° of elevation relative to the scapula—corresponding to approximately 0°, 45°, and 90° relative to the trunk—while maintaining maximal external rotation and horizontal extension. At each position, the contact area of the glenoid was demarcated on the articular cartilage of the humeral head (Fig. 2). The glenoid translated from the inferomedial to the superolateral aspect of the humeral head along the posterior margin of the articular surface, creating a distinct zone of contact. This trajectory was defined as the 'glenoid track.'

To accurately map the glenoid track, establishing its medial margin was of paramount importance, given that this specific boundary dictates the threshold for Hill-Sachs engagement. The distance from the medial margin of the rotator cuff footprint to the medial margin of the glenoid track was measured, which was determined to be 84% of the glenoid width.

**Fig. 3:**
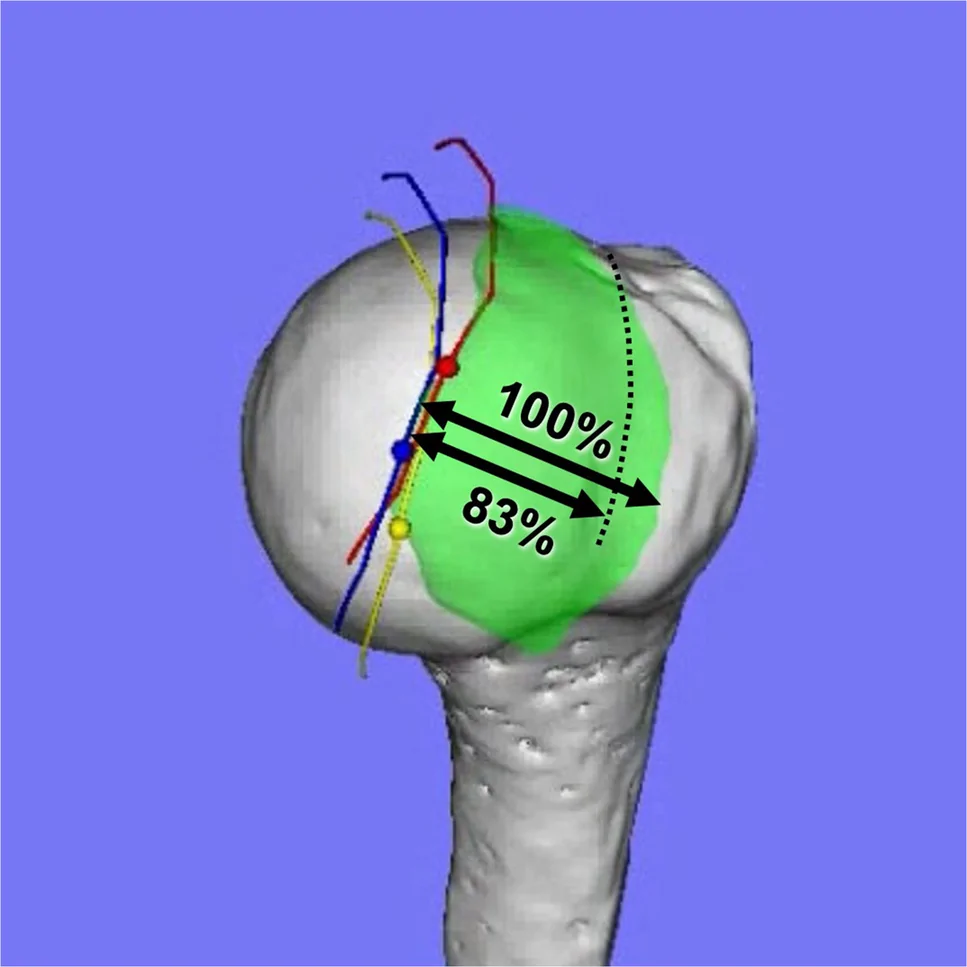
Glenoid track in live shoulders. The three lines indicate the movement of the anterior portion of the glenoid. The dotted line indicates the medial margin of the footprint of the rotator cuff. The glenoid track width was 83% of the glenoid width with the arm at 90° of abduction. (Reprinted from Fig. 5.16, Itoi [22])

Subsequently, we evaluated healthy volunteers to determine the location of the glenoid track in vivo. Using a wide-gantry MRI scanner, the right shoulders of 30 healthy volunteers were examined [24]. The arm was secured in seven static supine positions ranging from 0° to maximal abduction. Three-dimensional (3D) models of the glenoid and the humeral head were reconstructed, and the glenoid was projected onto the humeral head. The width of the glenoid track with the arm at 90° of abduction corresponded to 83% of the glenoid width (Fig. 3). This 83% threshold is currently used as the glenoid track width in daily practice.

## Clinical application

**Fig. 4:**
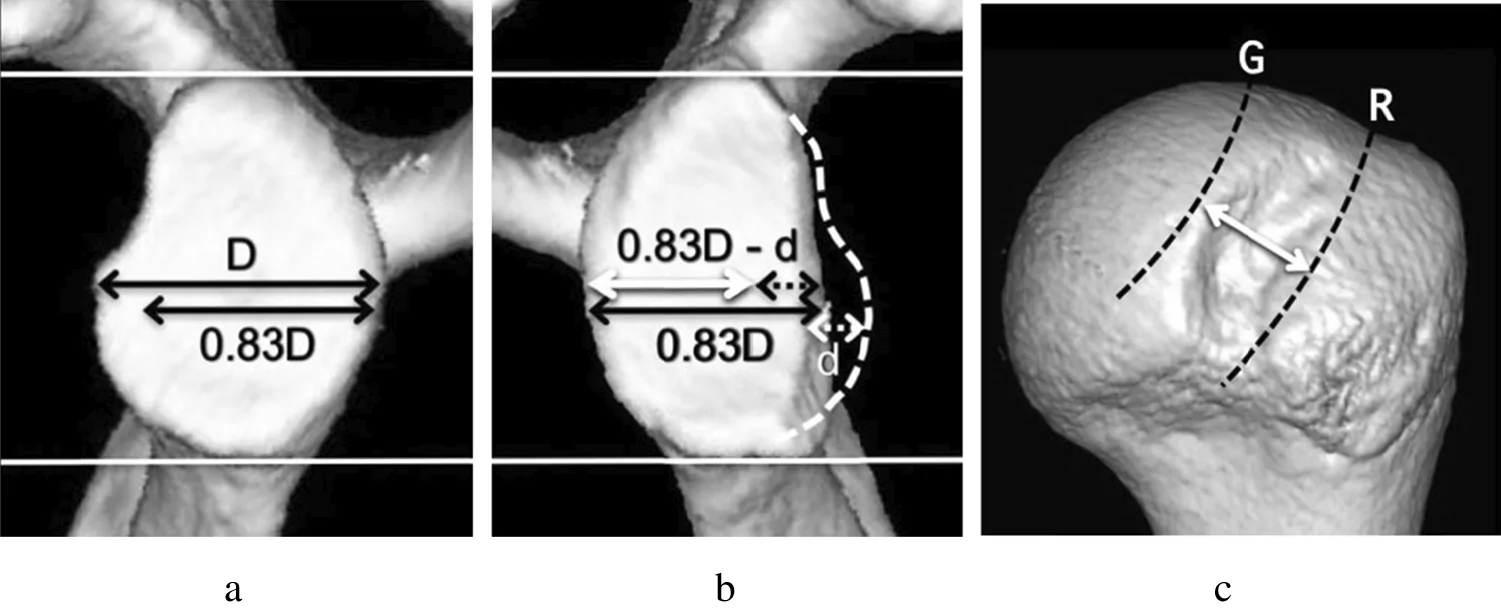
Assessment of on-track/off-track lesion. **a** In the intact contralateral shoulder, the total glenoid width (D) is measured. The standard glenoid track width is calculated as 83% of this value (0.83D). **b** In the affected shoulder, if glenoid bone loss is absent, 0.83D is utilized as the glenoid track width. If glenoid bone loss is present, a mirror-image profile of the contralateral glenoid is superimposed (white dotted line). The defect width (**d**) is then subtracted from 0.83D to determine the true glenoid track width (0.83D – d). **c** The true glenoid track width is subsequently projected onto the posterior aspect of the humeral head. The dotted line 'G' designates the medial margin of the glenoid track, whereas the dotted line 'R' indicates the medial margin of the rotator cuff footprint. In the case presented, the Hill-Sachs lesion remains entirely within the boundaries of the glenoid track, yielding a diagnosis of an 'on-track' lesion. (Reproduced with modification from Fig. 18, Itoi [13])

To facilitate widespread clinical adoption, the glenoid track concept required a nomenclature that was both attractive and clear. The task of naming Hill-Sachs lesions based on their position relative to the track's medial margin was undertaken by Giovanni Di Giacomo, Stephen S. Burkhart, and Eiji Itoi. While Di Giacomo initially suggested 'bone-buttress' and 'non-bone-buttress' lesions, our consensus shifted toward a system that directly integrated the glenoid track concept itself. Steve Burkhart then proposed the elegant terminology of 'on-track' and 'off-track' lesions. Recognizing its brilliance and clarity, we immediately adopted this naming scheme. Consequently, this clinical framework was introduced to the orthopaedic community in 2014 [25]. The current standard workflow for assessing the glenoid track using 3D CT imaging is delineated in Fig. 4.

Based on the glenoid track concept, all shoulder instability cases can be stratified into four distinct groups: Group 1, glenoid defect < 25% of the glenoid width combined with an on-track Hill-Sachs lesion; Group 2, glenoid defect < 25% with an off-track lesion; Group 3, glenoid defect ≥ 25% with an on-track lesion; and Group 4, glenoid defect ≥ 25% with an off-track lesion. Our recommended surgical treatment paradigm is tailored to each cohort: arthroscopic Bankart repair for Group 1; arthroscopic Bankart repair supplemented with remplissage for Group 2; the Latarjet procedure for Group 3; and the Latarjet procedure combined with either remplissage or Hill-Sachs bone grafting for Group 4. Notably, while the Latarjet procedure is also effective for Group 2, it is insufficient as a standalone option for Group 4. In Group 4, the coracoid process graft lacks adequate dimensions to convert an off-track Hill-Sachs lesion into an on-track lesion, regardless of the technique used. Consequently, viable surgical options in these severe cases entail either addressing the humeral defect directly via bone grafting or remplissage, or widening the glenoid track itself using an iliac crest bone graft.

Several technical limitations currently constrain the precise measurement of the glenoid track. First, estimating the original glenoid width in the setting of an anterior bone loss is inherently difficult, as the native anatomy is destroyed. While both the best-fit circle and contralateral comparison methods are clinically established, our data indicate that the contralateral comparison technique exhibits superior reliability for quantifying glenoid deficiency [26]. Second, defining the medial boundary of the rotator cuff footprint remains problematic on standard 3D CT. Although MRI excels at delineating soft-tissue attachments, current MR-based protocols are hindered by structural limitations; specifically, the exclusive availability of axial planes frequently exaggerates the Hill-Sachs interval, culminating in the overdiagnosis of off-track lesions [27]. Consequently, acquiring MR planes perpendicular to the long axis of the Hill-Sachs lesion is critical for an accurate bipolar bone loss classification. Further advancements in customized MR methodologies are currently underway to overcome these imaging limitations.

## Modern refinements

Currently, the standard clinical benchmark for the glenoid track width is established at 83% of the glenoid width. However, this fixed threshold merely represents an average, whereas the true glenoid track width dynamically fluctuates in accordance with an individual’s range of motion. In a patient with restricted range of motion—particularly in horizontal extension—the glenoid does not approximate the medial margin of the rotator cuff footprint during abduction, external rotation, and horizontal extension, thereby rendering the glenoid track width wider than the 83% baseline. Conversely, in individuals with a hyperlax shoulder demonstrating a broad range of motion, the glenoid overrides the rotator cuff tendon insertion, which substantially diminishes the glenoid track width.

Our previous cadaveric investigation validated this kinematics, demonstrating that the glenoid track width decreases linearly with increasing horizontal extension [28]. We subsequently evaluated this relationship in healthy volunteers and revealed that the active horizontal extension angle measured in the sitting position served as the most reliable predictor of the glenoid track width for each individual [29]. This specific correlation is defined by the following equation:

$${\boldsymbol{Y}}=-0.49{\boldsymbol{X}}+90$$

where ***X*** (°) represents the active horizontal extension angle in the sitting position, and ***Y*** represents the customized glenoid track width (%). This predictive model allows for the transition from a universal fixed standard to a truly personalized, patient-tailored glenoid track paradigm in clinical practice.

**Fig. 5:**
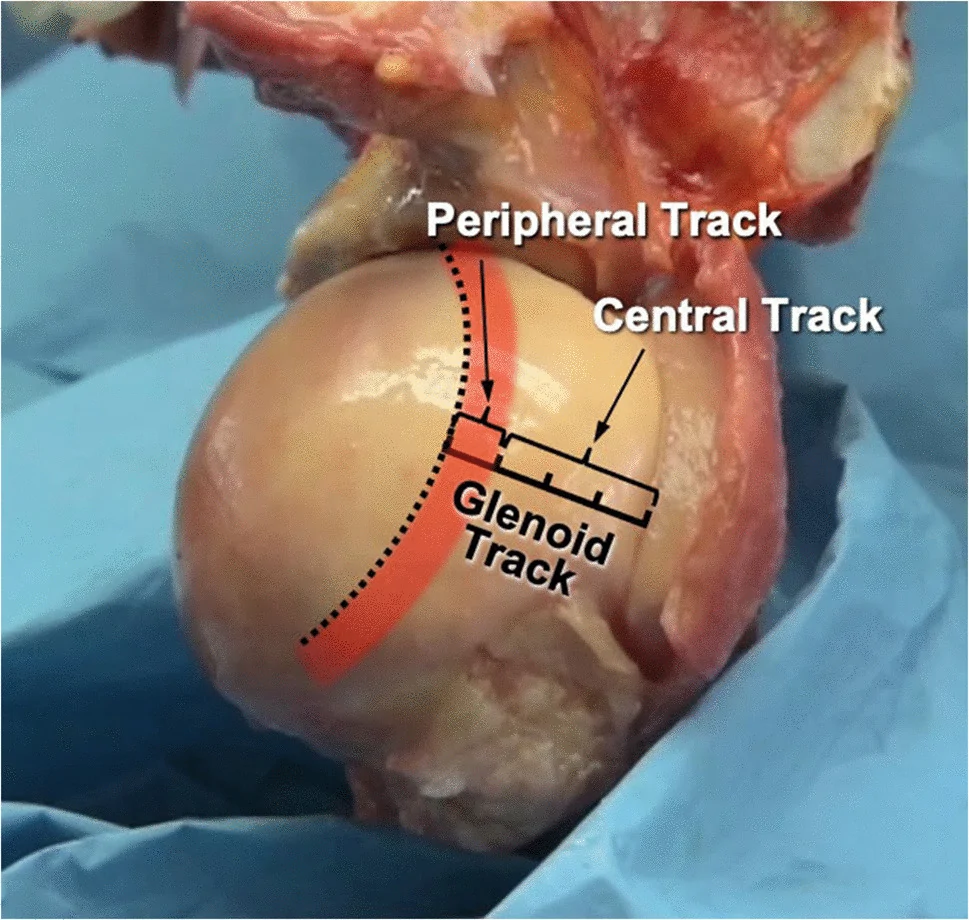
Peripheral track and central track. The most medial 1/4 of the glenoid track is called the peripheral track and the rest 3/4 the central track. The WOSI score was significantly worse if the Hill-Sachs lesion extends medially into the peripheral track. (Reprinted from Fig. 4, Itoi [31])

Parallel to the ambiguous demarcation between critical and safe glenoid defects characterized by subcritical bone loss, the glenoid track similarly exhibits a transitional 'gray zone' near its medial periphery. To investigate this boundary effect, we partitioned the glenoid track into four equal quadrants and compared the Western Ontario Shoulder Instability Index (WOSI) scores among them. Patients whose Hill-Sachs lesions extended medially into the most medial quadrant demonstrated significantly inferior WOSI scores. Based on these clinical findings, we designated this highly vulnerable medial quarter as the 'peripheral track' and the remaining three-quarters as the 'central track' [30] (Fig. 5). Consequently, patients presenting with a peripheral track lesion may warrant aggressive management comparable to an off-track lesion, particularly when they belong to a high-risk demographic.

## Future directions

While the glenoid track concept was originally introduced to assess the engagement risk of Hill-Sachs lesions in anterior shoulder instability, this paradigm can also be applied to posterior instability, which involves concomitant reverse Bankart and reverse Hill-Sachs lesions. Due to its relative rarity compared to anterior instability, a definitive consensus regarding surgical indications for posterior instability has yet to be established. Structurally, posterior instability is characterized by bony defects localized at the posteroinferior aspect of the glenoid—corresponding to the 8:12 o'clock position—with the trajectory of dislocation directed accordingly [32], while the companion reverse Hill-Sachs lesion is situated on the anterior aspect of the humeral head [33].

Adapting the methodology established by Omori et al. for anterior instability [24], we performed MRI on 20 shoulders from ten healthy volunteers across three standardized positions combining internal rotation and horizontal flexion at 0°, 45°, and 90° of shoulder flexion [34]. 3D models of the glenoid and humeral head were subsequently reconstructed, allowing the glenoid surface to be projected onto the humeral head to record its excursion across the anterior humeral aspect. This specialized path of articulation was designated as the 'reverse glenoid track.'

The width of the reverse glenoid track was quantified using two distinct anatomical references: the medial margin of the subscapularis footprint and the anterior margin of the humeral articular cartilage surface. The resulting track width ranged from 80 to 92% when measured from the subscapularis footprint, and from 50 to 59% when measured from the anterior articular margin. This novel 'reverse glenoid track' framework provides a critical tool for determining which specific lesions require surgical intervention. Parallel to the tenets of anterior instability, features such as subcritical glenoid bone loss, central versus peripheral track subdivisions, and personalized track-width adjustments based on patient-specific range of motion likely exist in posterior instability as well. Further investigation is warranted to precisely define these parameters and optimize the clinical utility of this concept in daily practice.

## Data Availability

No datasets were generated or analysed during the current study.

## References

[1] Kurokawa D, Yamamoto N, Nagamoto H, Omori Y, Tanaka M, Sano H, Itoi E (2013) The prevalence of a large Hill-Sachs lesion that needs to be treated. J Shoulder Elbow Surg 22:1285–1289. 10.1016/j.jse.2012.12.033 23466174

[2] Nakagawa S, Ozaki R, Take Y, Iuchi R, Mae T (2015) Relationship between glenoid defects and Hill-Sachs lesions in shoulders with traumatic anterior instability. Am J Sports Med 43:2763–2773. 10.1177/0363546515597668 26316609

[3] Sugaya H, Moriishi J, Dohi M, Kon Y, Tsuchiya A (2003) Glenoid rim morphology in recurrent anterior glenohumeral instability. J Bone Joint Surg Am. 10.2106/00004623-200305000-00016 12728039

[4] Etoh T, Yamamoto N, Shinagawa K, Hatta T, Itoi E (2020) Mechanism and patterns of bone loss in patients with anterior shoulder dislocation. J Shoulder Elbow Surg 29:1974–1980. 10.1016/j.jse.2020.03.022 32741565

[5] Itoi E, Lee SB, Berglund LJ, Berge LL, An KN (2000) The effect of a glenoid defect on anteroinferior stability of the shoulder after Bankart repair: a cadaveric study. J Bone Joint Surg Am 82:35–46 10653082 10.2106/00004623-200001000-00005

[6] Saito H, Itoi E, Sugaya H, Minagawa H, Yamamoto N, Tuoheti Y (2005) Location of the glenoid defect in shoulders with recurrent anterior dislocation. Am J Sports Med 33:889–893. 10.1177/0363546504271521 15827355

[7] Yamamoto N, Itoi E, Abe H, Kikuchi K, Seki N, Minagawa H, Tuoheti Y (2009) Effect of an anterior glenoid defect on anterior shoulder stability: a cadaveric study. Am J Sports Med 37:949–954. 10.1177/0363546508330139 19261900

[8] Yamamoto N, Muraki T, Sperling JW, Steinmann SP, Cofield RH, Itoi E, An KN (2010) Stabilizing mechanism in bone-grafting of a large glenoid defect. J Bone Joint Surg Am 92:2059–2066. 10.2106/jbjs.i.00261 20810855

[9] Burkhart SS, De Beer JF (2000) Traumatic glenohumeral bone defects and their relationship to failure of arthroscopic Bankart repairs: significance of the inverted-pear glenoid and the humeral engaging Hill-Sachs lesion. Arthroscopy 16:677–694 11027751 10.1053/jars.2000.17715

[10] Burkhart SS, Debeer JF, Tehrany AM, Parten PM (2002) Quantifying glenoid bone loss arthroscopically in shoulder instability. Arthroscopy 18:488–491. 10.1053/jars.2002.32212 11987058

[11] Shaha JS, Cook JB, Song DJ, Rowles DJ, Bottoni CR, Shaha SH, Tokish JM (2015) Redefining “critical” bone loss in shoulder instability: functional outcomes worsen with “subcritical” bone loss. Am J Sports Med 43:1719–1725. 10.1177/0363546515578250 25883168

[12] Yamamoto N, Kawakami J, Hatta T, Itoi E (2019) Effect of subcritical glenoid bone loss on activities of daily living in patients with anterior shoulder instability. Orthop Traumatol Surg Res 105:1467–1470. 10.1016/j.otsr.2019.08.015 31624030

[13] Itoi E (2017) “On-track” and “off-track” shoulder lesions. EFORT Open Rev 2:343–351. 10.1302/2058-5241.2.170007 28932486 PMC5590004

[14] Rowe CR, Patel D, Southmayd WW (1978) The Bankart procedure: a long-term end-result study. J Bone Joint Surg Am 60:1–16 624747

[15] Buhler M, Gerber C (2002) Shoulder instability related to epileptic seizures. J Shoulder Elbow Surg 11:339–344. 10.1067/mse.2002.124524 12195251

[16] Kaar SG, Fening SD, Jones MH, Colbrunn RW, Miniaci A (2010) Effect of humeral head defect size on glenohumeral stability: a cadaveric study of simulated Hill-Sachs defects. Am J Sports Med 38:594–599. 10.1177/0363546509350295 20194958 PMC2836589

[17] Hardy P, Lopes R, Bauer T, Conso C, Gaudin P, Sanghavi S (2012) New quantitative measurement of the Hill-Sachs lesion: a prognostic factor for clinical results of arthroscopic glenohumeral stabilization. Eur J Orthop Surg Traumatol 22:541–547. 10.1007/s00590-011-0883-x

[18] Parke CS, Yoo JH, Cho NS, Rhee YG (2012) Arthroscopic remplissage for humeral defect in anterior shoulder instability: is it needed? Katakansetsu 37:333

[19] Cho SH, Cho NS, Rhee YG (2011) Preoperative analysis of the Hill-Sachs lesion in anterior shoulder instability: how to predict engagement of the lesion. Am J Sports Med 39:2389–2395. 10.1177/0363546511398644 21398576

[20] Haviv B, Mayo L, Biggs D (2011) Outcomes of arthroscopic “remplissage”: capsulotenodesis of the engaging large Hill-Sachs lesion. J Orthop Surg Res 6:29. 10.1186/1749-799x-6-29 21676226 PMC3130690

[21] Zhu YM, Lu Y, Zhang J, Shen JW, Jiang CY (2011) Arthroscopic Bankart repair combined with remplissage technique for the treatment of anterior shoulder instability with engaging Hill-Sachs lesion: a report of 49 cases with a minimum 2-year follow-up. Am J Sports Med 39:1640–1647. 10.1177/0363546511400018 21505080

[22] Itoi E (2023) Shoulderology: searching for “whys” related to the shoulder. Springer, Singapore

[23] Yamamoto N, Itoi E, Abe H, Minagawa H, Seki N, Shimada Y, Okada K (2007) Contact between the glenoid and the humeral head in abduction, external rotation, and horizontal extension: a new concept of glenoid track. J Shoulder Elbow Surg 16:649–656. 10.1016/j.jse.2006.12.012 17644006

[24] Omori Y, Yamamoto N, Koishi H, Futai K, Goto A, Sugamoto K, Itoi E (2014) Measurement of the glenoid track in vivo as investigated by 3-dimensional motion analysis using open MRI. Am J Sports Med 42:1290–1295. 10.1177/0363546514527406 24681398

[25] Di Giacomo G, Itoi E, Burkhart SS (2014) Evolving concept of bipolar bone loss and the Hill-Sachs lesion: from “engaging/non-engaging” lesion to “on-track/off-track” lesion. Arthroscopy 30:90–98. 10.1016/j.arthro.2013.10.004 24384275

[26] Kuberakani K, Aizawa K, Yamamoto N, Shinagawa K, Suzuki T, Hatta T, Kawakami J, Itoi E (2020) Comparison of best-fit circle versus contralateral comparison methods to quantify glenoid bone defect. J Shoulder Elbow Surg 29:502–507. 10.1016/j.jse.2019.07.027 31564576

[27] Kimura R, Yamamoto N, Arino A, Kawakami J, Nagamoto H, Aizawa T, Itoi E (2025) The conventional magnetic resonance method may overestimate off-track Hill-Sachs lesions. J Shoulder Elbow Surg 34:2678–2684. 10.1016/j.jse.2025.02.046 40185391

[28] Yamamoto N, Kawakami J, Nagamoto H, Shiota Y, Itoi E (2018) The relationship between the glenoid track and the range of shoulder motion: a cadaver study. Orthop Traumatol Surg Res 104:793–796. 10.1016/j.otsr.2017.12.006 29292122

[29] Kawakami J, Yamamoto N, Etoh T, Hatta T, Mineta M, Itoi E, Isawa R (2019) In vivo glenoid track width can be better predicted with the use of shoulder horizontal extension angle. Am J Sports Med 47:922–927. 10.1177/0363546519825629 30870033

[30] Yamamoto N, Shinagawa K, Hatta T, Itoi E (2020) Peripheral-track and central-track hill-sachs lesions: a new concept of assessing an on-track lesion. Am J Sports Med 48:33–38. 10.1177/0363546519886319 31756135

[31] Itoi E (2022) Chapter 43 opinion editorial—first-time shoulder dislocation: my approach. In: Matsen FA, Cordasco FA, Sperling JW, Lippitt SB (eds) Rockwood and Matsen’s the shoulder, 6th edn. Elsevier, Philadelphia, pp 635–638

[32] Yang K, Yamamoto N, Takahashi N, Kamijo H, Okamura K, Mihata T, Sugaya H, Funakoshi T, Arino A, Kawakami J, Aizawa T, Itoi E (2026) Location and size of the glenoid defect in patients with traumatic posterior shoulder instability. J Orthop Sci. 10.1016/j.jos.2025.12.013 41611612

[33] Yang K, Yamamoto N, Takahashi N, Kamijo H, Okamura K, Mihata T, Sugaya H, Funakoshi T, Atsushi A, Kawakami J, Aizawa T, Itoi E (2025) Location and size of the reverse Hill-Sachs lesion in patients with traumatic posterior shoulder instability. J Shoulder Elbow Surg 34:88–95. 10.1016/j.jse.2024.03.017 38642873

[34] Ishizu N, Yamamoto N, Koibuchi Y, Sasaki K, Arino A, Kimura R, Kawakami J, Aizawa T, Itoi E (2025) A new concept of reverse glenoid track to determine which reverse Hill-Sachs lesion should be treated. Am J Sports Med 53:2075–2083. 10.1177/03635465251346953 40574268

